# Harness Organoid Models for Virological Studies in Animals: A Cross-Species Perspective

**DOI:** 10.3389/fmicb.2021.725074

**Published:** 2021-09-16

**Authors:** Yongming Sang, Laura C. Miller, Rahul K. Nelli, Luis Gabriel Giménez-Lirola

**Affiliations:** ^1^Department of Agricultural and Environmental Sciences, College of Agriculture, Tennessee State University, Nashville, TN, United States; ^2^Virus and Prion Research Unit, National Animal Disease Center, United States Department of Agriculture, Agricultural Research Service, Ames, IA, United States; ^3^Department of Veterinary Diagnostic and Production Animal Medicine, College of Veterinary Medicine, Iowa State University, Ames, IA, United States

**Keywords:** organoid, viruses, disease modeling, domestic and wild animals, zoonosis

## Abstract

Animal models and cell culture *in vitro* are primarily used in virus and antiviral immune research. Whereas the limitation of these models to recapitulate the viral pathogenesis in humans has been made well aware, it is imperative to introduce more efficient systems to validate emerging viruses in both domestic and wild animals. Organoids ascribe to representative miniatures of organs (i.e., mini-organs), which are derived from three-dimensional culture of stem cells under respective differential conditions mimicking endogenous organogenetic niches. Organoids have broadened virological studies in the human context, particularly in recent uses for COVID19 research. This review examines the status and potential for cross-species applied organotypic culture in validating emerging animal, particularly zoonotic, viruses in domestic and wild animals.

## Organoids: The Culturable Mini-Organs in Humans and Animals

Organoids, also known as “mini-organs,” are three-dimensional (3D) constructs; these constructs differentiate from stem cells to recapitulate the cellular architecture and functionality of native organs (Kaushik et al., 2018; Lehmann et al., 2019; Kim et al., 2020). Using both embryonic pluripotent stem cells (PSCs) and somatic adult stem cells (ASCs), organoids have been generated by culturing stem cells under conditions mimicking the *in vivo* developmental and differential cues of their respective tissues (Kaushik et al., 2018; Lehmann et al., 2019; Corrò et al., 2020; Kim et al., 2020). The cornerstone of organoid systems are stem cells, and relevant research signifies the origin of organoid systems. Further elucidation of biochemical (e.g., growth factors and other bioactive molecules) and physical cues (e.g., mechanic stimuli and topography applied from the extracellular matrix) serves as a “cow pony,” shaping organoid culture to regenerate multi-cellular tissue proxies of original tissues and organs (Yin et al., 2016; Moghaddam et al., 2019; Corrò et al., 2020; Fuchs and Blau, 2020; Hofer and Lutolf, 2021).

### Comparative Advantages of Organoid Systems

As per the virus-host interactions, animal models and two-dimensional (2D) cell cultures represent major biological experimental systems in biomedical studies. Animal models directly replicate animal infections in nature, and most of these models holistically recapitulate human pathophysiology. However, they are limited by individual variations, inter-species biological differences, interference with other microorganisms (pathogenic or common flora), animal welfare and ethical concerns; obstacles in throughput and convenience for microimaging and analytic readouts are also present (Shanks et al., 2009; Mead and Karp, 2019; Corrò et al., 2020; Kim et al., 2020; Hofer and Lutolf, 2021). In contrast, 2D monocultures of cell lines offer a consistent and reproducible *in vitro* model supporting viral infections, and they are widely used in virus-cell interaction studies. However, cancerous cell lines are genetically instable and over-simplistic to recapitulate the complex inter-cell and cell-matrix interactions that are required to model viral pathogenesis and particularly antiviral reactions in tissues (Shanks et al., 2009; Mead and Karp, 2019; Corrò et al., 2020; Kim et al., 2020; Hofer and Lutolf, 2021). Different alternatives of cell culture systems including co-culture of cell lines or primary cells and cell aggregate cultures of targeted tissues have both shown improved features compared to monotype cell culture. However, they lose the relevance to tissue organization *in vivo*—and even cell integrity—due to 2D culture adaptation. In addition, tissue explant cultures—which require rushed handling after surgeries to transiently retain physiologic relevance of cell organization/interaction— undergo cell phenotypic loss and senescence in order to align with an adequate time-window for performing studies in virology and antiviral immunology (Shanks et al., 2009; Mead and Karp, 2019; Corrò et al., 2020; Schutgens and Clevers, 2020; Hofer and Lutolf, 2021). Hence, these model systems each exhibit a meaningful gap to recapitulate the cellular organization and physiology of original organs; this is especially true for studying virus-host interactions, which generally involve more than one cell type in the targeted tissues (Barrila et al., 2018; Ramani et al., 2018; Bar-Ephraim et al., 2020; Sridhar et al., 2020; Yuki et al., 2020). Upon thorough examination of recent advent and peer reviews in both humans and animals organoid research, it is clear that an urgent need has emerged to harness better biological systems; a balance between practicability and faithfulness is needed to assess the cross-species potential of viral pathogens in both humans and animals (Shanks et al., 2009; Barrila et al., 2018; Ramani et al., 2018; Montes-Olivas et al., 2019; Bar-Ephraim et al., 2020; Corrò et al., 2020; Sridhar et al., 2020; Yuki et al., 2020). The status and perspective of organoid systems has been particularly reviewed, demonstrating potency and comparative advantages in order to fulfill most needs for *in vitro* modeling of viral infections and pathogenesis in humans (Barrila et al., 2018; Ramani et al., 2018; Lehmann et al., 2019; Montes-Olivas et al., 2019; Bar-Ephraim et al., 2020; Kim et al., 2020; Sridhar et al., 2020; Yuki et al., 2020). Table 1 summarizes the pros and cons of 3D organoid cultures; they are compared with classical 2D cell cultures and animal models regarding their complexity, cost, controllability, and downstream biomedical applications (Shanks et al., 2009; Yin et al., 2016; Kaushik et al., 2018; Lehmann et al., 2019; Mead and Karp, 2019; Moghaddam et al., 2019; Corrò et al., 2020; Fuchs and Blau, 2020; Kim et al., 2020; Hofer and Lutolf, 2021). As each type of model generally possesses inherent peculiarities, increasing the scale entails higher system complexity and challenges for the success of controlled cultures; hence, a reduced accessibility emerges when analyzing approaches at molecular and cellular levels (Lehmann et al., 2019; Kim et al., 2020). Organoid models render a platform to coordinate moderate system complexity and reproducibility. It also simultaneously enables the probing of the structure and function of a given biological system. Even when compared with 3D cultures of tissue explants, organoid systems model better genetic stability and cell-cell/cell-matrix interactions; they also simultaneously permit for long-term cultures based on regeneration from stem cells and maintenance of biological cues (Yin et al., 2016; Ramani et al., 2018; Mead and Karp, 2019; Fuchs and Blau, 2020). Organoids bridge the gap between 2D cell culture and animal models by providing a culturable system; this allows manipulation of cell cultures while representing better mimicking of *in vivo* cellularity and physiology (Lehmann et al., 2019; Kim et al., 2020). As implicated in several pilot studies and eminent reviews, we emphasize that cross-species investigation of organoid cultures in domestic and wild animals, will significantly promote virology studies, particularly for efficiently validating the infection loop of epizootic and zoonotic viruses in different animal species, including humans (Barrila et al., 2018; Ramani et al., 2018; Lehmann et al., 2019; Montes-Olivas et al., 2019; Bar-Ephraim et al., 2020; Duque-Correa et al., 2020; Kim et al., 2020; Schutgens and Clevers, 2020; Sridhar et al., 2020; Yuki et al., 2020; Hofer and Lutolf, 2021; Kar et al., 2021; Pain, 2021).

**TABLE 1:** Comparison of 3D organoid culture with conventional cell cultures and animal models (Shanks et al., 2009; Yin et al., 2016; Kaushik et al., 2018; Lehmann et al., 2019; Mead and Karp, 2019; Moghaddam et al., 2019; Corrò et al., 2020; Duque-Correa et al., 2020; Fuchs and Blau, 2020; Kim et al., 2020; Hofer and Lutolf, 2021; Kar et al., 2021; Pain, 2021).

	2D cell culture	3D organoid culture	Animal models
Complexity	Low	Medium	High
Cost	Low	Low to medium	High
Manipulability and reproducibility	More uniformly controlled	Good, but may have more variability	Limited due to individual variation
Genome stability and biobanking	Genome instability	Yes	Yes at cellular level
Genome/gene editing	Yes	Yes	Yes, only using embryonic stem cells
Physiologic recapitulation	Limited	Semiphysiologic	Physiologic
Vascularization and system integration	No	Partial	Yes
Modeling organogenesis	Poor	Effective in modeling intra-organ cell-cell interaction and core morphogenesis	Yes, but often complicated by organismal complex
Modeling development and diseases in Animals	Poor, due to over-simplicity vs. animal body system	Yes, at organ level	Yes, genuine self if use same species
Modeling development and diseases in humans	Poor, due to over-simplicity and species-specificity	Partially, critical to model and study shared diseases and zoonotic infections	Yes, comparable holistic model

### The Status of Organoids and Application in Research of Animal Viruses

**FIGURE 1:**
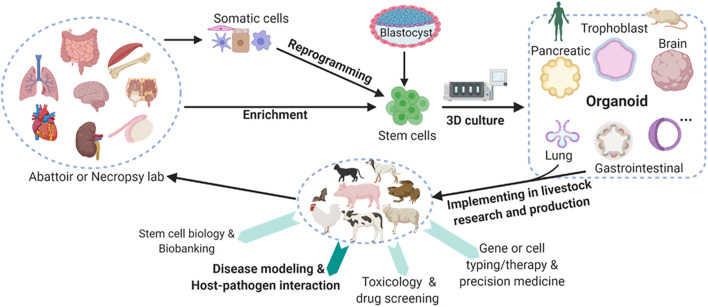
Schematic of mainstream organoid culture in humans and mice and their multifaceted potential in the promotion of domestic and wild animals studies. Organoids, also known as “mini-organs,” are three-dimensional (3D) constructs that differentiate from stem cells to recapitulate cellular architecture and functionality of native organs. Multiple types of organoids have been cultured primarily in humans and mice, and they have profoundly revolutionized the research and practice in human medicine. Some types of organoid culture have also been validated in livestock species. However, these cultures must be extended for promoting research and production in agricultural animals. Collectively, organoid research in domestic animals will provide dual roles and dual benefits in promoting the wellbeing of both the animals and humans with several advantages. These advantages include reliable offal resources, easier handling regarding organ size when compared to that of a mouse, and more physiological similarity in modeling human organs. Animal organoid studies will reveal unknown species-specific prospects in terms of organ development, stem cell biology, mechanistic regulation at gene and cellular levels, and particularly disease modeling and host-pathogen interaction for epizootic and zoonotic viral infections. Created with BioRender.com.

Multiple types of organoids have been cultured primarily from human and mouse stem cells, and this has revolutionized the research and clinical practice in human medicine; this is true in terms of applications in biobanking, drug screening, genotyping (i.e., cell typing) for precision medicine, and disease modeling involving pathogenic infections (Figure 1; Yin et al., 2016; Kaushik et al., 2018; Lehmann et al., 2019; Moghaddam et al., 2019; Corrò et al., 2020; Fuchs and Blau, 2020; Kim et al., 2020; Hofer and Lutolf, 2021). As demonstrated from a variety of pioneering studies, organoid technology can be adopted in most vertebrate species to retain its strength in modeling animal diseases; this is particularly true in the case of the microbe-host interaction in infectious cases (Barrila et al., 2018; Ramani et al., 2018; Montes-Olivas et al., 2019; Bar-Ephraim et al., 2020; Duque-Correa et al., 2020; Schutgens and Clevers, 2020; Sridhar et al., 2020; Yuki et al., 2020). In this regard, tissue/organ resources are readily available for establishing organoid culture, particularly in livestock animals; this also addresses the concerns of animal welfare, reducing the number of animals that may succumb to experimentally pathogenic infections and disease modeling (Kar et al., 2021; Pain, 2021). Organoid-relevant studies have been boosted among animals in the last 10 years (Derricott et al., 2019; Corrò et al., 2020; Seeger, 2020; Beaumont et al., 2021; Bourdon et al., 2021; Kar et al., 2021; Pain, 2021), with multiple studies using organoids to promote COVID-19 investigation (Clevers, 2020; Monteil et al., 2020; Yang et al., 2020; Han et al., 2021). Despite some types of organoid culture having been validated in multiple animals—particularly domestic species—there is an overall lack of robust procedures and dependent resources to generate animal organoids (Derricott et al., 2019; Seeger, 2020; Beaumont et al., 2021; Bourdon et al., 2021; Kar et al., 2021; Pain, 2021). These forms of organoid research in animals are advantageous, and they provide dual roles and benefits in promoting the wellbeing of animals and humans. Advantages include more reliable offal resources than medical biopsy samples, easier handling regarding organ size as compared to mice, and more physiological similarity in modeling human organs (Derricott et al., 2019; Lehmann et al., 2019; Kim et al., 2020; Seeger, 2020; Beaumont et al., 2021; Bourdon et al., 2021; Kar et al., 2021; Pain, 2021). It is also worth noting that the genetic stability retained by stem cells make organoid systems a promising tool for genetic rescue of endangered animal species.

Nevertheless, animal organoid studies are also essential for modeling epizootic and zoonotic diseases that generally involve multiple cell types. Relevant studies can reveal unknown species-specific prospects regarding organ development, stem cell biology, host-microbe interaction, and mechanistic regulation at the gene and cellular levels (Figure 1; Montes-Olivas et al., 2019; Bar-Ephraim et al., 2020; Yuki et al., 2020). Table 2 summarizes the status of organoid research in animals with highlighted applications in studying viral diseases (Derricott et al., 2019; Seeger, 2020; Beaumont et al., 2021; Bourdon et al., 2021; Kar et al., 2021; Pain, 2021). Intestinal organoids have been replicated in major domestic animal species and horseshoe bats (Derricott et al., 2019; Seeger, 2020; Zhou et al., 2020; Beaumont et al., 2021). Some studies further utilized intestinal organoids to successfully model enteric viral infections in cats and pigs (Li et al., 2019a,b, 2020; Luo et al., 2020; Tekes et al., 2020; Yin et al., 2020). Organoids of multiple other organs have also been established. These include canine organoids from the skin (Wiener et al., 2018), prostate gland (Usui et al., 2017), urinary bladder (Elbadawy et al., 2019), kidney (Chen et al., 2019) and liver (Nantasanti et al., 2015), as well as porcine organoids from the esophagus submucosal gland (von Furstenberg et al., 2017), rectum (Adegbola et al., 2017), and testicle (Sakib et al., 2019; Vermeulen et al., 2019). It also includes those from the mammary gland, pancreatic duct, and endometrium in cattle (Martignani et al., 2018), sheep (Liu et al., 2020) and horse (Thompson et al., 2020), respectively. Notably, no respiratory or lung organoids have been reported in animals. However, they have been reported in humans. There are only a few studies of organoids in wild animals, which include two recent reports in the bat intestine and snake venom glands (Post et al., 2020; Zhou et al., 2020). There is an imperative need of respiratory/lung organoids to model respiratory viral infections in animals; for this reason, we expect an increase in studies pertaining to animal respiratory organoids from active research in COVID-19 and other zoonotic respiratory viruses (Miller et al., 2019; Sachs et al., 2019; Han et al., 2021).

**TABLE 2:** Organoid cultures validated in domestic and wild animal species (Bourdon et al., 2021; Kar et al., 2021; Pain, 2021).

Group/Species	Source tissues	Disease modeling?	References
**Domestic**			
*Canis lupus familiaris* (Dog)	Skin (Keratinocyte) Prostate gland Urinary bladder Kidney Intestine Liver	N/A Prostate cancer Bladder cancer N/A N/A Copper storage disease	[Bibr B129][Bibr B122][Bibr B30][Bibr B14][Bibr B86]; [Bibr B13][Bibr B77]
*Felis catus* (Cat)	Intestine Liver	Feline coronavirus (Tekes et al., 2020) Hepatic lipidosis/steatosis	Powell and Behnke, 2017; [Bibr B116][Bibr B52]; Haaker et al., 2020
*Gallus gallus* (Chicken)	Intestine	Protozoan (*T. gondii*) (Holthaus et al., 2021)	Pierzchalska et al., 2017; Powell and Behnke, 2017; Panek et al., 2018; Pierzchalska et al., 2018; Acharya et al., 2020; Holthaus et al., 2021
*Sus scrofa* (Pig)	Esophagus submucosal gland Intestine Rectum Testicular tissue	N/A *Lawsonia* infection (Resende et al., 2020) Enteric coronavirus (Li et al., 2019a,b, 2020; Luo et al., 2020; Yin et al., 2020) Protozoan (*T. gondii*) (Holthaus et al., 2021) Crohn disease NA	[Bibr B125][Bibr B51]; van der Hee et al., 2018; Li et al., 2019a,b, 2020; Liu et al., 2020; Resende et al., 2020; [Bibr B135][Bibr B2][Bibr B95]; Vermeulen et al., 2019
*Bos Taurus* (Cattle)	Intestine mammary gland	N/A N/A	Hamilton et al., 2018; Derricott et al., 2019; [Bibr B119][Bibr B67]
*Ovis aries* (Sheep)	Intestine pancreatic duct	N/A Copper toxicity	Powell and Behnke, 2017 Liu et al., 2020
*Equus caballus* (Horse)	Intestine endometrium	N/A N/A	Powell and Behnke, 2017; [Bibr B111][Bibr B117]
*Oryctolagus cuniculus* (Rabbit)	Intestine	Rabbit calicivirus, but not productive	Kardia et al., 2021
**Wild**			
*Rhinolophus sinicus (Horseshoe bat)*	Intestine	SARS-CoV2/COVID-19	Zhou et al., 2020
Nine snake species	Snake venom glands	N/A	Post et al., 2020
Dogfish shark	Rectal gland	N/A	Schwarz et al., 2015

## Historical Brief and Promising Applications of Organoid Cultures in Virological Studies

### Historical Brief of the Organoid System and Uses in Animal Virological Research

**FIGURE 2:**
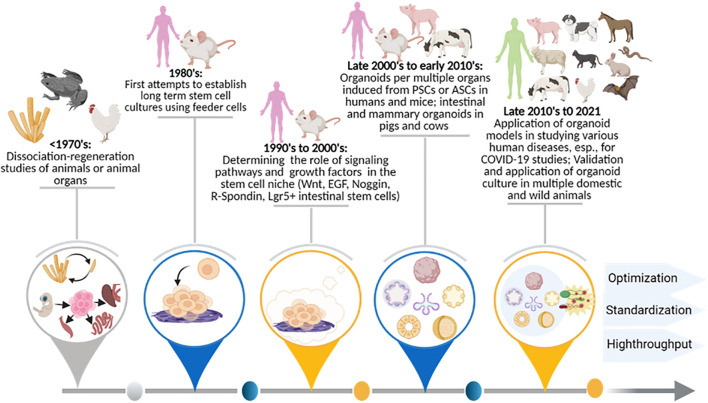
Historical stages for the development of organoid cultures, as well as the status and perspective for application in viral diseases in humans and animals. Summaries are shown of organoid research and technological advent benchmark breakthroughs in order to facilitate cross-species modeling of epizootic and zoonotic viral infections post-prospective development. The involvement of human and animal species are diagrammed at the top along with the major temporal events summarized below. ASC, adult stem cells; COVID-19, novel coronavirus disease 2019; EGF, epidermal growth factor; Lgr5+, Leucine Rich Repeat Containing G Protein-Coupled Receptor 5; Noggin, NOG gene product; PSC, pluripotent stem cell; R-Spondin, Rspo1 gene product; Wnt, Wingless and Int- signaling pathway.

Figure 2 illustrates major historical stages in the development of organoid cultures. The status and perspective to apply these to modeling viral diseases in humans, domestic animals, and wild animals is also shown. Foundational work regarding the observation and elucidation of stem cells’ role in tissue, organ, and organism regenerations dates back to the early last century; Dr. Henry Wilson scientifically demonstrated that dissociated sponge cells could self-organize to regenerate an entire organism *in vitro* (Wilson, 1907; Corrò et al., 2020). Later, similar dissociation-reaggregation experiments were successfully performed using dissociated amphibian pronephros (Holtfreter, 1944) and chick embryos (Weiss and Taylor, 1960) to generate different types of organs; this implies a differential adhesion hypothesis for stem cell-derived self-regeneration (Steinberg, 1964). Pioneering studies centered on stem cell handling began with two groups in 1981, when PSCs were first isolated and established from mouse embryos (Evans, 1981; Martin, 1981). Human PSCs were first isolated and cultured from human blastocysts in 1998 (Thomson et al., 1998; Corrò et al., 2020). Both mouse- and human-induced PSCs (iPSCs) were subsequently obtained by reprogramming fibroblasts or somatic cells in the mid-2000s; this burgeoned the research in both stem cell biology and application for organoid systems *in vitro* (Takahashi and Yamanaka, 2006; Takahashi et al., 2007; Yu et al., 2007).

Following the discovery of stem cells in the 1980s, studies began to boom in harnessing stem cell culture and differentiation; this was done by simulating relevant microenvironment cues *in vivo* (Corrò et al., 2020). Using extracellular matrix (ECM) reconstructed from an Engelbreth-Holm-Swarm tumor (EHS), Li et al. (1987) were able to maintain mammary epithelia growth and induce the formation of 3D milk-secreting duct-lumen structures; these structures would have otherwise rapidly lost their ability to secrete milk protein in the monocellular culture. Similarly, the presence of ECM of EHS also helped alveolar type II epithelial cells to retain their cell morphology; it helped them maintain function as compared to cells cultured on plastic dishes (Shannon et al., 1987). This highlights the vital role of both cell-cell and cell-matrix interactions in maintaining cell/tissue function *in vitro*. Eiraku et al. (2008) recapitulated early corticogenesis in order to generate cerebral cortex tissue from both human and mouse ESCs; this was done using a 3D aggregation culture method. Around the same time, Sato et al. (2009) identified adult intestinal stem cells as leucine-rich repeat-containing G protein-coupled receptor 5 (Lgr5)-expressing cells at the bottoms of small-intestinal crypts; they further established intestinal organoids through cultured single crypts or sorted Lgr5(+) stem cells in Matrigel (Sato et al., 2009). This study established 3D organoid culture from ASC-containing tissues or a single ASC; and in the late 2000s and early 2010s, this bookmarked the flourish of organoid studies in humans and various animal species (Kaushik et al., 2018; Lehmann et al., 2019; Corrò et al., 2020; Kim et al., 2020). Generated using either tissues that containing ASCs/PSCs or stem cells themselves, organoid systems have been successfully established in different organ systems, including the mesendoderm-type (e.g., stomach, liver, pancreas, lung, and kidney) and the neuroectoderm-type (brain and retina) in mice and humans (Figure 2; Kaushik et al., 2018; Lehmann et al., 2019; Corrò et al., 2020; Kim et al., 2020). The most recent success includes establishing organoid culture simulating snake venom glands for venom peptide production (Post et al., 2020) and application of various organoid systems for disease modeling; this is emphasized to efficiently model infections by viruses, a group of cell-obligated infectious agents challenging the classical 2D cell cultures (Barrila et al., 2018; Ramani et al., 2018; Sachs et al., 2019; Bar-Ephraim et al., 2020; Schutgens and Clevers, 2020; Sridhar et al., 2020; Yuki et al., 2020).

### Promising Applications of Organoid Cultures in Virological Studies

Animal models and cell cultures (including both primary cells and immortalized cell lines) are broadly used in most aspects of virological studies; this includes assays involving virus entry, titration, cytopathy, and rescue (Ramani et al., 2018; Schutgens and Clevers, 2020; Sridhar et al., 2020). However, these classical models present obvious limitations (Barrila et al., 2018; Ramani et al., 2018; Bar-Ephraim et al., 2020; Schutgens and Clevers, 2020; Sridhar et al., 2020; Yuki et al., 2020). These limitations are particularly related to physiological similarity and inter-cell interaction of natural hosts. Of the noted advantages, organoids have been used to extend virological studies in several aspects (Ramani et al., 2018; Schutgens and Clevers, 2020; Sridhar et al., 2020). First, organoids recapitulate viral infections more efficiently from naturally infected tissue samples in the laboratory. When compared to the general need for extensive screening and serial adaptation using cell cultures (especially cancerous cell lines from other species), organoids might support the replication of the wild-type virus when they originate from relevant organs where clinical isolates are surgically obtained (Ramani et al., 2018; Schutgens and Clevers, 2020; Sridhar et al., 2020). It has been shown that viruses from most infected human samples (e.g., nasopharyngeal swabs, blood, or feces) were more readily grown in organoids or airway epithelial cultures (Palmer et al., 2012; Tapparel et al., 2013; Bochkov et al., 2016; Sridhar et al., 2020). Animal viruses are often empirically cultured and studied using cell lines of primate or other species origins; and this is due to the unavailability or lack of support regarding cell cultures from the species of a viral natural host. For examples, swine arterivirus (Cafruny et al., 2006; Calvert et al., 2007) and coronaviruses were replicated and studied using cell lines from the monkey kidney (Hofmann and Wyler, 1988; Govorkova et al., 1996; [Bibr B58][Bibr B59], 2020; Luo et al., 2020; Yin et al., 2020) and MDCK, a canine kidney cell line used widely for studies of influenza virus from human and various animal resources (Takada et al., 2019). Given the species-tropism of viruses, extensive targeting is needed for adaption and mutation using animal models to replicate productive infections of human viruses; this kind of cross-species adaptation increases biosafety concerns (Simmonds et al., 2019; Takada et al., 2019; Dai et al., 2020; Kuchipudi et al., 2021). Nonetheless, viral tropism, receptor usage, and virus-cell interaction in these species-distinct cells or animal models may divert from genuine virus-host interaction in the natural host (Hofmann and Wyler, 1988; Govorkova et al., 1996; Simmonds et al., 2019; Takada et al., 2019; Dai et al., 2020; Kuchipudi et al., 2021).

Second, organoids enable the culture of viruses that are unculturable in cells. As with a number of virological study cases, it is futile to find suitable cell lines to replicate and rescue a wild-type virus that causes active infection *in vivo* (Leland and Ginocchio, 2007; Rabenau et al., 2007; Hodinka, 2013). Organoid systems constitute a convenient alternative for culturing these viruses under laboratory conditions (Ramani et al., 2018; Schutgens and Clevers, 2020; Sridhar et al., 2020). Despite extensive tests using available cell lines, viruses including human norovirus, human coronavirus HKU1, bocavirus, and rhinovirus C remain futile for productive replication; this is possibly due to certain viruses’ need for inter-cell or cell-matrix interaction for effective infection. Studies showed that human intestinal organoids supported norovirus and rotavirus infection; human airway epithelial cultures allowed culture and characterization of human coronaviruses HKU1 and NL63, human bocavirus, and human rhinovirus C, which implicated the potential of relevant respiratory organoids (Dijkman et al., 2009; Pyrc et al., 2010; Hao et al., 2012; Tapparel et al., 2013; Farsani et al., 2015). Therefore, multiple cell types differentiated in organoid systems provide updated culturable biological platforms, and these can be utilized for further virological characterization and rescuing of viruses from clinical isolates (Ramani et al., 2018; Schutgens and Clevers, 2020; Sridhar et al., 2020).

Third, organoid systems allow the determination of authentic virus-host interaction and recapitulation findings *in vivo*; this is still in its infancy in terms of virological application. Epithelia that line the skin, blood vessels (specified as endothelia), and internal tracts/glands of digestive and respiratory systems provide primary portals for virus infection initiation. In the body, epithelial cells are polarized as apical (oriented toward the lumen) and basolateral (oriented away from the lumen) sides with differential expression of various surface molecules; this can be exploited as entering receptors for viral infections (Bomsel and Alfsen, 2003; Compans and Herrler, 2005). Contrary to the loss or difference of surface molecules expressed in 2D-cultured cells and animal models of other species, organoids of relevant tissues reconstruct the cellular organization/polarization; this allows dissecting the polarized entry and an *in vitro* spread of viruses in the epithelial barrier (Bomsel and Alfsen, 2003; Compans and Herrler, 2005; Ramani et al., 2018; Schutgens and Clevers, 2020; Sridhar et al., 2020). The characterization of measles virus (MV) cellular receptors provides an eloquent example. This calls for increased use of “*vivo*” models—such as organoids—for characterizing the pathogenesis of wild-type viruses. Previous studies using vaccine- and laboratory-adapted MV strains and cell cultures resulted in the discovery of the MV cellular receptor as CD46. This was a cell-surface molecule widely expressed by nucleated cells. The discovery was based on *in vitro* findings induced by the thought that MV infection entered the apical side of respiratory epithelia (Dorig et al., 1993). Recent studies incorporating *ex vivo* and *in vivo* approaches determined nectin-4 as a genuine receptor of wild-type MV strains. Nectin-4 is restrictively expressed at the basolateral side of epithelial cells. This indicated that respiratory epithelial cells are infected later from the basolateral side post-epithelial damage. The virus is then released into the respiratory tract. This contrasted the previous proposal of MV initiating epithelial infection through its apical side (Ludlow et al., 2010; Laksono et al., 2020).

Organoid systems also potentially demonstrate clinical signs, as observed in viral infections of natural hosts. For instance, 5–14% of children born with Zika virus infections suffered severe neurological complications, including inducing microcephaly or abnormally small heads (Qian et al., 2017). Recently, this clinical observation was recapitulated using human-brain organoids derived from induced PSCs (iPSCs). It was shown that Zika virus infection reduced organoid size in mimicking the virus-caused microcephaly *in vivo*. Studies using human brain organoids also potentiated insight of neurotropism and pathogenesis of other neurotropic viruses; this including neonatal herpes simplex virus (HSV) and congenital-cytomegalovirus, causing broad neurological defects such as microcephaly (Wen et al., 2017; Brown et al., 2019; D’Aiuto et al., 2019; Depla et al., 2020; Sridhar et al., 2020). Studies using porcine enteroids—generated from different segments of the intestine—successfully recapitulated the segment- and cell-dependent tropism. Using porcine enteroids, the antiviral interferon (IFN) response as observed *in vivo* was demonstrated during infections with two porcine enteric coronaviruses, porcine epidemic diarrhea virus (PEDV) and transmissible gastroenteritis virus (TGEV) (Li et al., 2019a,b, 2020; Luo et al., 2020; Yin et al., 2020). In addition, the virus-organoid models can be readily applied to drug screening procedures, and this leads to effective therapeutic interventions for mitigating viral infection (Ramani et al., 2018; Depla et al., 2020; Schutgens and Clevers, 2020; Sridhar et al., 2020; Figure 1).

Fourth, organoids extend the capacity of a culture system to support replication of multiple viruses (including pathogens of other phyla) that have different cell tropisms. This allows for studying ecological interaction among co-infected agents and with the host systems. While these kinds of co-infection models better reflect the complexity of natural infections, they are increasingly necessary. An updated characterization is also need in regards to the microbiome’s contribution in shaping viral infections and antiviral immunity. For example, human enteroids showed susceptibility to infection with several enteroviruses, including echovirus 11 (E11), coxsackievirus B (CVB), and enterovirus 71 (EV71). However, they depended on different cell lineages—contained by the enteroids—to induce virus-specific antiviral and inflammatory responses. Enteroids therefore provide an ecological hub for characterization of virus-specific pathogenesis; this also includes antiviral responses with the presence of interacting microbes (Sridhar et al., 2020). The use of organoids to model viral co-infections promotes better understanding and therapeutic interventions for syndromes or disease complexes; these are centered by one to several etiological viruses commonly observed in the digestive, respiratory, and reproductive tracts (Kim et al., 2020; Min et al., 2020; Sridhar et al., 2020; Hofer and Lutolf, 2021).

## Organoids Provide a Valuable Model for Cross-Species Validating Emerging Animal and Zoonotic Viruses

### Multi-Organ Types of Organoids Used in Studying COVID-19

Over a dozen types of organoids have been successfully established per human organs, including the brain, intestine, kidney, liver, lung, and pancreas (Kaushik et al., 2018; Lehmann et al., 2019; Corrò et al., 2020; Kim et al., 2020). Most of these human organoids were also applied in disease modeling; several of these were piloted to study diseases caused by viruses, including human rotavirus, coronaviruses HKU1 and VL63, bocavirus, rhinovirus C, measles virus, enteroviruses, and the Zika virus (Ramani et al., 2018; Schutgens and Clevers, 2020; Sridhar et al., 2020). The outbreak and ongoing pandemic of the new coronavirus 2019 disease (COVID-19) drove the wide application of organoids; this was done to recapitulate the disease and validate its virological insights of severe acute respiratory syndrome coronavirus 2 (SARS-CoV-2) (Clevers, 2020; Sridhar et al., 2020). The lung organoids—or organoid-like alveolar epithelia (HAE)—containing specified airway ciliated cells are productive in supporting SARS-CoV-2 replication, as clinically observed *in vivo* (Sridhar et al., 2020; Yang et al., 2020; Han et al., 2021). The respiratory organoids and the HAE model also demonstrated virus-caused epithelial damage and death processes underlying the plaque-like cytopathies observed in the lung of COVID-19 pneumonia (Milewska et al., 2020; Yang et al., 2020; Han et al., 2021). For therapeutic evaluation, Pizzorno et al. (2020) showed that remdesivir and remdesivir–diltiazem were effective against SARS-CoV-2 infection in both nasal and bronchial epithelia cultures (Pizzorno et al., 2020; Sridhar et al., 2020).

SARS-CoV-2 transmission among humans occurs principally through exposure to virus-containing respiratory fluids (Clevers, 2020; Han et al., 2021). It must be noted that effective transmission may occur through mucous membranes in the mouth, nose, or eyes. This can happen as a result of either passive deposition or active inhalation. This indicates that the epithelial lining of these body organs are generally susceptible to the virus. After initial infection in the airway epithelia, SARS-CoV-2 may spread systemically and cause dysfunction in multiple organs such as the gut, liver, kidney, testicle, and brain. Vasculitis or endothelitis ascribes inflammation of blood vessels or vascular cells (endothelia), which consist of a major systemic symptom underlying most organ-specific manifestations in COVID-19 patients. Therefore, COVID-19 was proposed to be a vascular disease, and this suggests that SARS-CoV-2 causes direct endothelial infection/injury while mediating multi-system dysfunction (Siddiqi et al., 2021). Penninger et al. established both capillary organoids and kidney organoids from human iPSCs, and they demonstrated that SARS-CoV-2 could directly infect endothelial cells therein (Monteil et al., 2020). These findings explain the systemic spread of the virus; it also explains renal dysfunction in patients with severe COVID-19 (Monteil et al., 2020; Siddiqi et al., 2021). The potential of SARS-CoV-2 to cause secondary infections in multiple organs was also demonstrated using organoid models. Several studies showed productive infection of SARS-CoV-2 in human enteroids or intestinal organoids; and demonstrated the sentinel role of Type III interferons (IFNs)—a group of epithelia-specific IFNs—in controlling the viral infection at an early phase (Lamers et al., 2020; Stanifer et al., 2020). Findings implicated direct gut infection and potential of fecal transmission of SARS-CoV-2, and rationalized the prolonging detection of SARS-CoV-2 in fecal samples during clinical screening of convalescent patients (Clevers, 2020; Sridhar et al., 2020; Wu et al., 2020).

Neurological manifestations occur in about 36.4% of COVID-19 patients. Relevant complications include loss of smell, headaches, ischemic stroke, muscle weakness, and encephalitis. Neuropathic pain has even been found as a post-COVID-19 symptom. These observations have promoted the use of brain organoids for assessing potential SARS-CoV-2 neuro-invasion. Two studies demonstrated that SARS-CoV-2 targeted neuron cells to cause direct infection of human brain organoids (Pellegrini et al., 2020; Ramani et al., 2020; Zhang et al., 2020). Using choroid plexus organoids, Pellegrini et al. (2020) demonstrated that SARS-CoV-2 was capable of causing brain infection through targeting the network of brain blood vessels; this explained the presence of the virus in the cerebrospinal fluid (CSF) and other parts of the brain. Therefore, studies using tissue-specific organoids of the human brain provide eloquent evidence regarding neurological complications of SARS-CoV-2 infections, as clinically observed in a portion of COVID-19 patients (Clevers, 2020; Pellegrini et al., 2020; Ramani et al., 2020; Sridhar et al., 2020; Zhang et al., 2020). Further studies increasing throughput using patient-originated organoids may critically address the broad diversity of COVID-19 manifestations as well as the association with various preexisting comorbidities in the patients (Clevers, 2020; Sridhar et al., 2020).

### Organoids Provide a Valuable System for Cross-Species Virological Research

**FIGURE 3:**
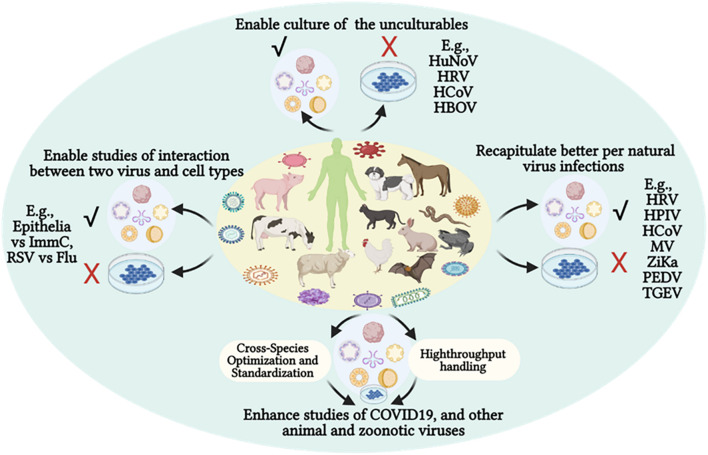
Organoid culture potentiates virological studies, providing a better system to enable recapitulating of the natural viral infections regarding productive culture (√) of previous unculturable viruses using cell lines (X) and mimicking *in vivo* clinical signs and cell interactions. The advantages of organoid cultures in virological studies have recently been demonstrated using multiple human viruses—particularly in COVID-19 studies—and have shaped prospective cross-species modeling of epizootic or zoonotic viral infections. COVID-19, new coronavirus disease 2019; Flu, influenza virus; HBOV, human bocavirus; HCoV, endemic human respiratory coronaviruses; HPIV, human parainfluenza virus; HuNoV, human norovirus; HRV, human rhinovirus; ImmC, immune cells; MV, measles virus; PEDV, porcine epidemic diarrhea virus; TGEV, porcine transmissible gastroenteritis coronavirus; RSV, respiratory syncytial virus; Zika, Zika virus.

SARS-CoV-2 seems to have a zoonotic origin evolutionarily from relevant coronaviruses in horseshoe bats; however, there is no scientific consensus about certain intermediate host(s) to bridge SARS-CoV-2 transmission to human beings (Conceicao et al., 2020; Sang et al., 2020, 2021). Remarkably, several animal species—including dogs, cats, minks, ferrets, hamsters, lions, and tigers—of both domestic and wild groups have been shown to be infected by SARS-CoV-2 through a zooanthroponotic (or reverse-zoonotic) way (Conceicao et al., 2020; Sang et al., 2020, 2021). It is impractical to experimentally validate viral susceptibility in most relevant animals, especially in wild species; however, organoid cultures and epidemiological prediction can be integrated to handle this task, especially after essential optimization from the established human systems (Figure 3; Ramani et al., 2018; Schutgens and Clevers, 2020; Sridhar et al., 2020). In this regard, Zhou et al. (2020) established intestinal organoids from a China horseshoe bat species using the culture condition for the human counterpart. As expected, the bat intestinal organoids were readily infected by SARS-CoV-2, in contrast to abortive trials using cell cultures (Zhou et al., 2020).

Along with emerging virome studies, viruses of known and unknown families were profiled from animal fecal samples as well as other animal or environmental samples at the genome level (Cadwell, 2015; Ramani et al., 2018; Wang, 2020). Nonetheless, most of these viruses are incapable of replication using cell culture or even laboratory animal models (Cadwell, 2015; Wang, 2020). This bat and other animal organoid studies are promising indications that culture conditions for human organoids should generally be applicable for most mammalian—and even vertebrate—species (Table 2; Derricott et al., 2019; Seeger, 2020; Zhou et al., 2020; Beaumont et al., 2021; Bourdon et al., 2021; Kar et al., 2021; Pain, 2021). Cross-species development of comparable organoid cultures will provide an effective biological system for validating the zoonotic (and reverse zoonotic) potential of newly emerging viruses. This potentiates cross-species assessment of virus-susceptibility in both domestic and wild animals in order to prevent zooanthroponotic and re-emerging zoonotic cycles (Conceicao et al., 2020; Sang et al., 2020, 2021). Moreover, organoids generated from animal organs—aligned with their respective virome studies—will allow culturing of novel viruses that are difficult to culture in established cell lines (Cadwell, 2015; Ramani et al., 2018; Moghaddam et al., 2019; Wang, 2020).

Infection tests in animals provide direct evidence for cross-species and species-specific infectivity of emerging animal and zoonotic viruses. Limitations of animal models owe to individual diversity and concerns on animal welfare. However, organoid systems offer a great substitute to document cross-species and species-different infectivity of targeted viruses (Sridhar et al., 2020; Hofer and Lutolf, 2021). In this regard, studies using human organoids have proven to be efficient prospects for investigating the species-specific susceptibility of influenza viruses. Influenza viruses—particularly the type A subfamily (IAV)—infect a broad range of vertebrate species; they also present a paradigm for studying emerging viruses that possess both epizootic and zoonotic threats (Long et al., 2019; Hofer and Lutolf, 2021). Human airway organoids from iPSCs contained the major airway epithelial cells types including ciliated cells, goblet cells, club cells, and basal cells. When applied to the modeling of IAV infections, these airway organoids provided discernment of different infectivity of emerging flu viruses, particularly those of avian or human origin. As shown in one comparative study, human airway organoids behaved similarly to the *ex vivo* cultured bronchus explants; this regarded tissue/cell tropism, virus production, and cytokine response to either human or avian IAV strains (Long et al., 2019; Sridhar et al., 2020; Hofer and Lutolf, 2021). With the procedural development for successful cryopreservation, subculture, and imaging resolution, human organoids have the potential to examine zoonotic threats of influenza and other emerging viruses (Wiley et al., 2016; Tsai et al., 2018; Dekkers et al., 2019; Tysoe et al., 2019). An estimation of over 60% of known infectious diseases in humans are zoonotic in origin; prominent diseases have the organ tropism for the respiratory or digestive systems, and can even span multiple systems (Jones et al., 2008). Most organoid cultures validated in animals are of the intestinal type (Table 2). Urgent and increasing public concerns for studying emerging animal and zoonotic viruses should be considered; it is imperative to adapt human organoid technology and develop animal organoid systems for respiratory and other organ types (Yin et al., 2016; Kaushik et al., 2018; Lehmann et al., 2019; Miller et al., 2019; Corrò et al., 2020; Fuchs and Blau, 2020; Kim et al., 2020). However, further challenges for the application of organoids in virological studies await substantial species-specific optimization and cross-species standardization to harness organoid cultures in a high-throughput platform (Figure 3).

## Challenges and Future Perspectives for Cross-Species Organoids Use in Virological Studies

### Species-Specific Optimization and Characterization

Until recently, studies of stem cell biology and organoid systems have been performed primarily in humans and mice (Yin et al., 2016; Kaushik et al., 2018; Lehmann et al., 2019; Corrò et al., 2020; Fuchs and Blau, 2020; Kim et al., 2020). Despite practices for establishing intestinal and other organoids in most domestic and few wild animal species (Table 2), species-specific optimization of culture conditions is required for maintaining and differentiating organoids (Derricott et al., 2019; Seeger, 2020; Beaumont et al., 2021; Bourdon et al., 2021; Kar et al., 2021; Pain, 2021). Even for the generation of human or mouse intestinal organoids, empirically conditioned media are used to provide essential growth factors including Wnt3a, Noggin, and R-spondin; these factors foster the growth and differentiation of stem cells into organotypic structures (Yin et al., 2016; Kaushik et al., 2018; Lehmann et al., 2019; Moghaddam et al., 2019; Corrò et al., 2020; Fuchs and Blau, 2020; Kim et al., 2020; Hofer and Lutolf, 2021). This introduces uncertainties for reproducing some organoid cultures in different laboratories within the same species (Sridhar et al., 2020; Hofer and Lutolf, 2021). Most growth factors for organoid cultures can be specified using recombinant proteins from reliable commercial suppliers. However, a recent study using side-by-side comparison indicated that Wnt3a-conditioned media was more supportive for the long-term survival of human or mouse colon organoids (Wilson et al., 2021). In addition, the availability of relevant recombinant proteins is questionable for most animal species (Dawson et al., 2020). Hence, in organoid cultures of another species, the use of growth factors (and conditioned media) with a human- or mouse-origin requires a substantial optimization process; it may even work better at different dosages or regimens (Shanks et al., 2009; Yin et al., 2016; Kaushik et al., 2018; Lehmann et al., 2019; Mead and Karp, 2019; Moghaddam et al., 2019; Corrò et al., 2020; Dawson et al., 2020; Fuchs and Blau, 2020; Kim et al., 2020; Hofer and Lutolf, 2021).

After establishing an organoid culture, it is subjected to characterization— particularly for cell heterogeneity and lineage differentiation (gene expression)—in mimicking respective organ proxies in addition to dynamically monitoring the morphology and growth property of the organoid. This requires immunoreagents to determine various cell markers; these have been historically developed in humans and mice and are very limited for most animal species (Faldyna et al., 2007; Sopp et al., 2007; Dawson et al., 2020). If no cross-reactive immunoreagents can be identified from the available resources, it is necessary to target the development of these species-specific immunoreagents and growth factors; this provides the basis for the authentic characterization of animal organoids (Faldyna et al., 2007; Sopp et al., 2007; Dawson et al., 2020). Because of the ultimate goal—namely, usage in virological studies—intensive molecular and cellular characterization may be performed in parallel or secondary to virus infection tests; this can determine functional potential in virological research (Ramani et al., 2018; Schutgens and Clevers, 2020; Sridhar et al., 2020).

### Cross-Species Comparability and Harmonization

Variation inherited from starting materials and conditioned media generated by different laboratories introduce challenges, particularly for cross-species standardizing of a given type of organoid culture, or even solely in humans (Kim et al., 2020; Sridhar et al., 2020; Hofer and Lutolf, 2021). Previous studies have shown that human intestinal organoids—derived in different medium formulations or using different starting tissues—had different levels of marker gene expression and cellular composition (Kim et al., 2020; Sridhar et al., 2020; Hofer and Lutolf, 2021). More applicable criteria and guidelines may be needed for cross-species organoid cultures to reduce variability; however, the supportive fact for a given virus infection in the organoids should be a major consideration, provided the major tissue proxy is recapitulated (Ramani et al., 2018; Schutgens and Clevers, 2020; Sridhar et al., 2020). In this context, a harmonized system for cross-species culture of organoids is proposed for modeling animal infectious diseases. Holthaus et al. (2021) generated intestinal organoids for four species—human, mouse, pig, and chicken—which are common hosts of *Apicomplexa* and other protozoa of zoonotic concern. This study unified the culture of intestinal organoids from different species into a comparable system for studying the protozoan infections. In addition, they provided straightforward guidelines and a simple medium formulation for cross-species generation of intestinal organoids from respective intestinal crypts. Using the organoid-derived monolayer culture in a Transwell system, they demonstrated the suitability of this system to support the protozoan infections as well as the potential to study parasite-host interactions for relevant intestinal protozoan co-infections (Holthaus et al., 2021). Currently, no virological studies have reported a form of comparison using cross-species organoids; however, it warrants relevant studies to determine cross-species susceptibility of emerging animal and zoonotic viruses (Ramani et al., 2018; Schutgens and Clevers, 2020; Sridhar et al., 2020; Holthaus et al., 2021).

### Increasing Throughput

Adequate scale-up defines the maturity of organoid systems that target cross-species validation of viral infections and antiviral responses. Aside from essential species-specific optimization and inter-species comparison, increasing throughput of handling comprises a further challenge for efficient use of organoid technology, particularly in screening of virus susceptibility and therapeutic antivirals (Kim et al., 2020; Sridhar et al., 2020; Hofer and Lutolf, 2021). This must be arranged to master resource suppliers like American Tissue and Cell Culture (ATCC); it must also unify standards for quality control in regards to the complex nature of the organoid systems. In addition to the existing commercial settings and multiplex formats developed primarily for cell cultures, the invention and utilization of multiplex culture formats/scaffolds are needed for culturing and analyzing organoids. This kind of high-throughput adaptation will make organoid application compatible with extensive omics technology, facilitating studies on system virology that target the goal of one-health initiative (Kim et al., 2020; Sridhar et al., 2020; Hofer and Lutolf, 2021).

### Inclusion of Immune and/or Microbiota Niches

Most organoids mainly recapitulate a single-organ system representing the epithelial capsule of specified functions (Yin et al., 2016; Kaushik et al., 2018; Lehmann et al., 2019; Moghaddam et al., 2019; Corrò et al., 2020; Fuchs and Blau, 2020; Kim et al., 2020; Hofer and Lutolf, 2021). For instance, gut organoids comprise the villus-bearing enterocytes, goblet cells, and enteroendocrine cells. Lung organoids contain ciliated cells, club cells, goblet cells, and basal cells. In general, organoids (with the exception of organoids for lymph nodes or tonsils) lack the mesenchymal or immune-cell niches that are integrated with their respective organs (Kim et al., 2020; Sridhar et al., 2020; Hofer and Lutolf, 2021). This prevents studying inter-system reactions that are commonly observed in antiviral responses involving interacting parts *in vivo* (Bar-Ephraim et al., 2020; Yuki et al., 2020). Tissue stromal cells and especially immune cells—such as macrophages for porcine arterivirus and bovine viral diarrhea virus—commonly serve as susceptible cells to define a virus tropism (Sang et al., 2015). Lack of these supportive cells in organoids may result in non-productive infection and virological modeling failure (Sang et al., 2015; Bar-Ephraim et al., 2020; Yuki et al., 2020). Both tissue stromal cells and immune cells function in secreting cytokines to shape the local microenvironment niche and potentiate antiviral immunity; this, in turn, affects the susceptible status of epithelial cells in the organoids (Sang et al., 2015; Bar-Ephraim et al., 2020; Yuki et al., 2020). This may provide a rationale for the inability to infect rabbit enteroids using a rabbit poxvirus (Kardia et al., 2021). Several approaches have been proposed to include stromal and immune cells in organoids. First, epithelial organoids may be co-cultured with tissue stromal cells, immune cells (including macrophages, dendritic cells, and T cells), or even the cell population from organoids of blood vessels or lymph nodes. Using this co-culture system, several studies have successfully demonstrated the interaction of epithelial cells and immune cells in antiviral reactions (Miranda et al., 2018; Holloway et al., 2019; Duzagac et al., 2021; Hofer and Lutolf, 2021). Unlike their ASC-derived counterparts, PSC-derived epithelial organoids may contain a mesenchymal layer, as shown in some human and mouse studies. Most animal organoids were ASC-derived due to the lack of specific PSCs and the comparative convenience of culture handling over the PSC-dependent protocol (Derricott et al., 2019; Seeger, 2020; Beaumont et al., 2021; Bourdon et al., 2021; Hofer and Lutolf, 2021; Kar et al., 2021; Pain, 2021). Second, the organ-on-a-chip system can be used to incorporate different organotypic functions. The organ-on-a-chip system is based on a reductionist engineering approach to culture major tissue cells; this uses a 3D microfluidic cell culture chip to capture key functions of an organ (Miranda et al., 2018; Holloway et al., 2019; Duzagac et al., 2021). Different types of organoids provide functional organ components in order to model the complexity of viral pathogenesis and antiviral immunity at a multi-system level. This multi-system organization of organoids may be suitable for investigating immunometabolic and immunoneurologic interaction underlying antiviral regulation (Bar-Ephraim et al., 2020; Yuki et al., 2020; Hofer and Lutolf, 2021). Third, multi-organ organoids—or organoids that include different physiological niches—can be developed through advanced organoid engineering technology (Min et al., 2020; Hofer and Lutolf, 2021).

The microbiota represents another functional niche coating both internal organ tracts and external skin; this plays a critical role in propelling pathogenic infections and training antiviral immunity (Rooks and Garrett, 2016; Li et al., 2019c). Due to the sterile condition of the stem cell and organoid cultures and the complexity of animal microbiota, it is not practical to include even most commensal microbes (including viruses that are numerously prominent there) during organotypic cultures. Recent studies have shown that immune education by microbiota can be rebuilt; this can be done by introducing only one to few representative microbes in the gut of germ-free mice. It was proposed that *Escherichia coli, Helicobacter pylori*, or rotavirus might be included to recapitulate the immune education role of microbiota in intestinal organoids (Min et al., 2020).

### Other Technological Challenges of Current Organoid Systems and Potential Approaches to Improve Them for Virological Application

Despite its success and potential as demonstrated, both organoid technology and its application in disease modeling are still in infancy (Shanks et al., 2009; Yin et al., 2016; Kaushik et al., 2018; Ramani et al., 2018; Lehmann et al., 2019; Mead and Karp, 2019; Moghaddam et al., 2019; Corrò et al., 2020; Fuchs and Blau, 2020; Kim et al., 2020; Schutgens and Clevers, 2020; Sridhar et al., 2020; Hofer and Lutolf, 2021). Table 3 lists major technological limitations/challenges and relevant resolutions of current organoid systems in virological applications. In summary, one inherent limitation stems from the short lifespan of most organoids (especially those generated from various organ ASCs) and the consequential immaturity compared to the adult organs *in vivo*. Given the need for modeling virus infections at the adult and even senescent status, some physical or biochemical stimuli may be required to induce maturation of most organoids (Kim et al., 2020; Sridhar et al., 2020; Hofer and Lutolf, 2021). The newly developed protocol for cryopreservation then sub-passage of an established organoid may physically elongate the duration, particularly for using the “colonized” organoid and increasing reproducibility of relevant tests (Wiley et al., 2016; Tsai et al., 2018; Dekkers et al., 2019; Tysoe et al., 2019). Otherwise, virus adaption may be necessary for a provided organoid system in a relatively juvenile state. Secondly, most organoids may form encapsulated epithelial structures within the enclosed lumen and lumen-facing apical side. This causes a limited exchange of nutrients and waste removal from the lumen side. It requires special handling (such as injection or physical poration) to introduce viruses inside the lumen, as most epithelia-tropic viruses infect from the apical side (Sridhar et al., 2020; Hofer and Lutolf, 2021). An alternative is to culture organoid cells to form 2D cell layers in a Transwell system; this will be analogical to the classical 2D culture for efficient cell exposure to virus infection and facilitate downstream optical analyses—among others—based on cells (Wiley et al., 2016; Tsai et al., 2018; Dekkers et al., 2019; Tysoe et al., 2019). In addition, a recent study developed an apical-out intestinal organoid in pigs; this was used to successfully model the infection of porcine TGEV and study local interferon responses (Li et al., 2020). Finally, the recent application of engineering principles to organoid technology enables inter-system incorporation (such as vascular systems, immune cells, and microbiota niches); it also increases experimental robustness, which is applicable to promoting organoid uses for virus research (Miranda et al., 2018; Holloway et al., 2019; Sridhar et al., 2020; Duzagac et al., 2021; Hofer and Lutolf, 2021).

**TABLE 3:** Limitations of current organoid systems and potential solutions for virus research (Shanks et al., 2009; Lehmann et al., 2019; Kim et al., 2020; Hofer and Lutolf, 2021).

Limitations	Organoid type	Points for prospective solutions
Limited week-scale lifespan	All, esp., ASC-derived	•Cryopreservation •Virus adaption •Organoid engineering
Maturation	All, esp., PSC-derived	•Maturation handling •Virus adaption •ASC-derived usage
Lack cell/system complexity	ASC-derived epithelial types	•Usage of PSC-derived to differentiate mesenchymal layer •Co-culture of cells/other organoids •Introduction of microbiota niche
Inaccessibility	Encapsulated organoids	•Increasing nutrient circulation through methods such as shaking and pumping •Cryopreserving at thriving status •Introduction of virus *via* injections or other physical poration •Develop apical-out epithelial organoids •Adopt 2D Transwell culture for simple cell layer
Limited reproducibility	Esp., ASC-derived	•Standardize resources for scaffolds and medium supplies •Standardize and simplify protocol •Use well-defined start materials for ASC-derived, and colonized PSC stocks •Subculture organoids from a cryopreserved organoid stocks
Readout limitations	Aggregated organoid capsules	•Imaging: (1) Use organoid microscopy or imager; (2) Adopt 2D Transwell culture; (3) Make organoids into continual sections •Molecular monitoring: implement biosensors •Computational modeling simulation •Omics profiling: Connect to various OMICS platforms, especially updates based on single cell throughput

## Concluding Remarks

Organoid systems utilize the self-organizing properties of stem cells to model multi-cellular proxies of organ tissues. Possessing properties intermediate between conventional cell culture and animal models, organoids have gained enormous interest for modeling disease, personalized medicine, drug screening, and organ therapy in humans (Shanks et al., 2009; Yin et al., 2016; Kaushik et al., 2018; Ramani et al., 2018; Lehmann et al., 2019; Mead and Karp, 2019; Moghaddam et al., 2019; Corrò et al., 2020; Fuchs and Blau, 2020; Kim et al., 2020; Schutgens and Clevers, 2020; Hofer and Lutolf, 2021). There are limitations presented in cell culture and animal models to recapitulate viral pathogenesis and host antiviral responses; for this reason, organoid systems are attracting attention in emerging, authentically-validating animal and zoonotic viruses across animals and humans (Ramani et al., 2018; Schutgens and Clevers, 2020; Sridhar et al., 2020). Organoids have already broadened virological studies in the human context, particularly with recent use in COVID-19 research (Clevers, 2020; Sridhar et al., 2020). Despite considerable success in modeling viral infections, limitations and challenges remain in virological studies involving emerging animal and zoonotic viruses (Ramani et al., 2018; Clevers, 2020; Schutgens and Clevers, 2020; Sridhar et al., 2020). Further studies are needed to standardize culture protocols, apply engineering principles, and increase both the reproducibility and utility in virological research and therapies (Kim et al., 2020; Hofer and Lutolf, 2021; Holthaus et al., 2021).

## Author Contributions

YS conducted overall conceptualization, reference collection and process, digestion, draft writing and finalization, and funding acquisition. LM, RN, and LG-L contributed to the conception, discussion, and proofreading. All authors contributed to the article and approved the submitted version.

## Conflict of Interest

The authors declare that the research was conducted in the absence of any commercial or financial relationships that could be construed as a potential conflict of interest.

## Publisher’s Note

All claims expressed in this article are solely those of the authors and do not necessarily represent those of their affiliated organizations, or those of the publisher, the editors and the reviewers. Any product that may be evaluated in this article, or claim that may be made by its manufacturer, is not guaranteed or endorsed by the publisher.
